# Myth or truth: investigation of the jumping ability of *Tunga penetrans* (Siphonaptera: Tungidae)

**DOI:** 10.1093/jme/tjad143

**Published:** 2023-10-20

**Authors:** Ayako Hyuga, Paul Ouma, Abneel K Matharu, Jürgen Krücken, Satoshi Kaneko, Kensuke Goto, Ulrike Fillinger

**Affiliations:** Human Health Theme, International Centre of Insect Physiology and Ecology, Nairobi 00100, Kenya; Human Health Theme, International Centre of Insect Physiology and Ecology, Nairobi 00100, Kenya; Human Health Theme, International Centre of Insect Physiology and Ecology, Nairobi 00100, Kenya; Institute for Parasitology and Tropical Veterinary Medicine, Freie Universität Berlin, Robert-von-Ostertag-Str. 7, 14163 Berlin, Germany; Institute for Parasitology and Tropical Veterinary Medicine, Freie Universität Berlin, Robert-von-Ostertag-Str. 7, 14163 Berlin, Germany; Department of Eco-Epidemiology, Institute of Tropical Medicine, Nagasaki University, 1-12-4 Sakamoto, Nagasaki-shi 852-8523, Nagasaki, Japan; Division of Health and Safety Sciences Education, Department of Educational Collaboration, Osaka Kyoiku University, 4-698-1 Asahigaoka, Kashiwara-shi 582-8582, Osaka, Japan; Human Health Theme, International Centre of Insect Physiology and Ecology, Nairobi 00100, Kenya

**Keywords:** *Tunga penetrans*, tungiasis, parasitic skin disease, jumping ability

## Abstract

Female sand fleas (*Tunga penetrans* Linnaeus, 1758, Siphonaptera: Tungidae) cause a severe parasitic skin disease known as tungiasis. *T. penetrans* is a small flea, measuring less than 1 mm in length. The females of this species burrow into the skin of human and animal hosts and mostly affect the feet. This has led to the anecdotal assumption that *T. penetrans*, unlike its relatives in the Siphonaptera family, would have a limited jumping ability potentially not reaching higher body parts. However, there is no data supporting this. This study evaluated the jumping capabilities of *T. penetrans* for height and distance using sticky tapes. The vertical jump of the female *T. penetrans* ranged from 4.5 to 100 mm with a mean of 40 mm whereas the vertical jump of the male *T. penetrans* ranged from 1.2 to 138 mm with a mean of 46 mm. The horizontal jump of the female *T. penetrans* ranged from 18 to 138 mm with a mean of 64 mm and that of the male ranged from 9 to 251 mm with a mean of 80 mm. Based on the literature, fleas of various species have been described as jumping vertically 50–100 times their size and horizontally 5–100 times their size. In this respect, sand fleas appear to have equal expert jumping abilities to their relatives. Their aggregation on people’s feet is not likely a result of their poor jumping ability but might be an adaptation to the host’s behavior which would require further investigations.

## Introduction

**Fig. 1.**
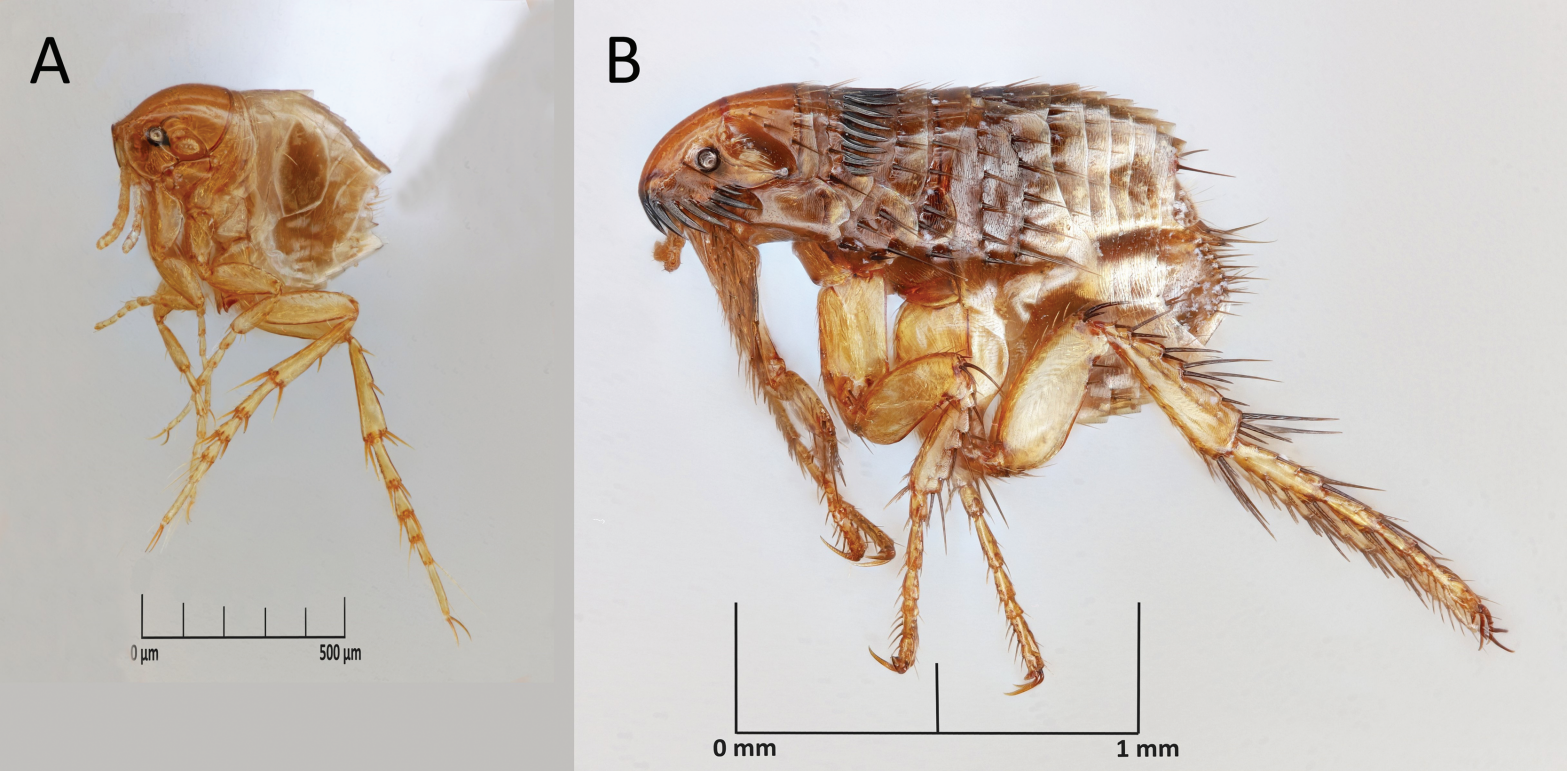
a) *Tunga penetrans* (female); b) *Ctenocephalides felis* (female) for size comparison. Scale adjusted.

Tungiasis is a neglected tropical disease characterized by a zoonotic skin epidermis parasitism of female sand fleas, *Tunga penetrans* (Linnaeus, 1758) (Siphonaptera: Tungidae) and *Tunga trimamillata* which inflicts pain and itching, and often results in secondary infections and walking difficulties due to manipulations in an attempt to remove the parasites. The disease affects primarily people living under poor conditions in tropical areas (Feldmeier et al. 2014). While female sand fleas burrow into the skin and remain permanently attached to blood-feed on the host, male sand fleas live freely on the host, feeding on blood and mating with the embedded females (Witt et al. 2004, Thielecke and Feldmeier 2013, Elson et al. 2022). The skin-embedded parasite is majorly found in the feet of its human and animal hosts (Heukelbach et al. 2002, Nagy et al. 2007). This observation has led to a statement in the literature: “Because the chigoe [sand fleas] cannot jump well, the feet are the preferred site for infestation” (Burke et al. 1991). Similar statements seem to have been copied across articles and websites (Heukelbach et al. 2001, 2002, Klimpel et al. 2005, Sachse et al. 2007, Ngan 2008, Pampiglione et al. 2009, Thomas 2022). This statement appears to have its origin in historic reports on tungiasis dating back to the 18th and 19th centuries when the disease was first described (Waterton and Moore 1871, Hoeppli 1963). However, *Tunga* spp., which measure less than 1 mm and are among the smallest fleas in the world (Fig. 1), have never been systematically studied for their jumping ability. To further our knowledge of the parasite’s behavior and ecology and to gauge biosafety measures required for laboratory experiments, we tested the vertical and horizontal jumping ability of freshly emerged adult sand fleas. Here, we focused on *T. penetrans*, which is distributed in sub-Saharan Africa as well as South America and the Caribbean (Heukelbach et al. 2001). *T. trimamillata* has been reported only from South America (Pampiglione et al. 2002, 2003, Fioravanti et al. 2003).

## Materials and Methods

The experiments were conducted at the International Centre of Insect Physiology and Ecology (icipe), Muhaka Field Station in Msambweni sub-county, Kwale County, Coastal Kenya. Soil samples for isolating off-host *T. penetrans* flea stages were collected from tungiasis-infested households in the surrounding villages. Samples were collected from sleeping areas of tungiasis-infected members in the context of field sampling activities of larger risk factor surveys prepared for publication elsewhere. The soil samples were exposed to heat using a Berlese Tullgren funnel (Amugune et al. 2022). *Tunga* larvae were identified morphologically under a microscope (Hicks 1930, Nagy et al. 2007) and their identity was confirmed through PCR-based methods (Amugune et al. 2022). Larvae were gently transferred to a larval medium, a mixture of a pinch of fish food (Tropical Flake Fish Food, Supa Aquatic Supplies Ltd., Sheffield, UK) and soil, and reared till pupation at ambient conditions at 24.2–29.0°C, natural light and at a relative humidity of 60%–99%. Soil substrates were prior to use sterilized in a pressure cooker for an hour.

**Fig. 2.**
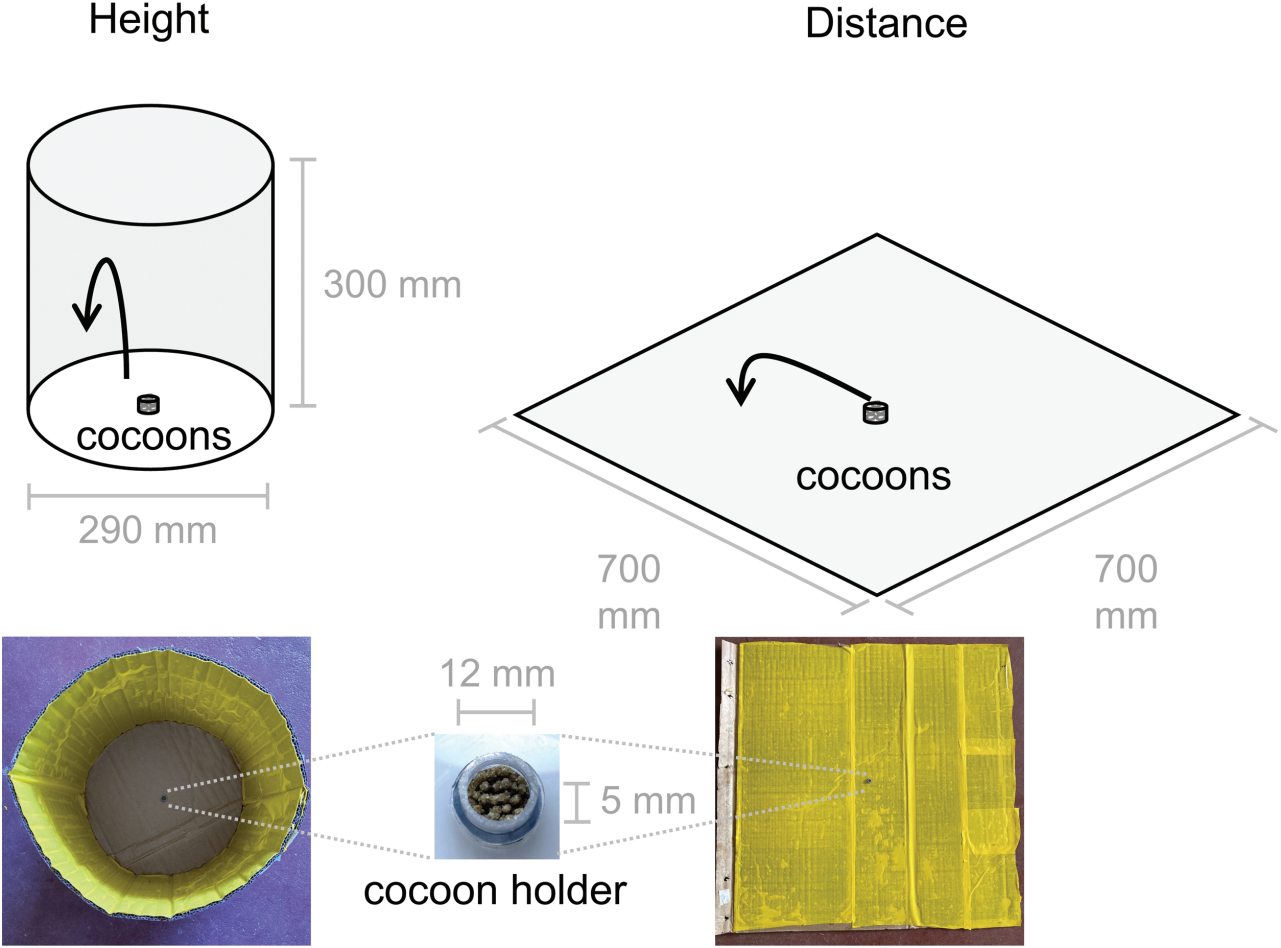
Measurement apparatuses. Sticky tape was located around walls of cylinder for height measures and across flat surface for distance. Arrows indicate the expected trajectory of the jump.

The pupae cocoons were introduced into 2 systems to measure the adults’ vertical and horizontal jumping performance. Preliminary test informed the design and size of the test arenas and served to confirm that the sicky material was sufficiently strong to hold the fleas in place. For vertical measurements, we constructed a cylindrical apparatus from cardboard with a diameter of 290 mm and a height of 300 mm. Prior to introducing the cocoons in the apparatus, we attached sticky tape (Koppert Biological Systems Inc., Berkel en Rodenrijs, Netherlands) to the inside walls of the apparatus to record the jumping height of emerged adults (Fig. 2). We placed a cocoon holder cut from the top part of a 1.5 ml centrifuge tube with a diameter of 12 mm and a height of 5 mm, containing 121 *Tunga* cocoons, at the center inside the cylindrical measurement apparatus, and closed the top of the apparatus with sticky tape. The cocoon holder was made as small as possible while holding more than a hundred cocoons. The apparatus was kept at ambient conditions until all adults had emerged and jumped or died inside the cocoon, which took 2–3 weeks. The height was measured for each flea stuck on the tape from the bottom with a ruler. For measuring the horizontal jumping distance, flat cardboard (700 mm long and 700 mm wide) covered with sticky tape was used as a jumping arena (Fig. 2) following procedures used for investigating dog and cat fleas (Cadiergues et al. 2000). A total of 119 *Tunga* cocoons were tested using the same cocoon holder. After 2 weeks, adults had either emerged and jumped or died in cocoons. The jumping distance was measured for each flea from the center point to the area where it was stuck. Flea species and sex were individually confirmed under a stereo microscope (20× magnification) (Smit 1973, Nagy et al. 2007).

R version 4.2.2 was used for statistical analyses and visualization. Welch’s t-test was used to compare jumping performance between female and male fleas. The statistical significance level was set to 0.05.

The study was conducted according to the guidelines of the Declaration of Helsinki and approved by the Scientific and Ethics Review Unit of Kenya Medical Research Institute (NON-KEMRI 644). Prior to sampling of soil, written informed consent was sought from the head of the household.

## Results

**Fig. 3.**
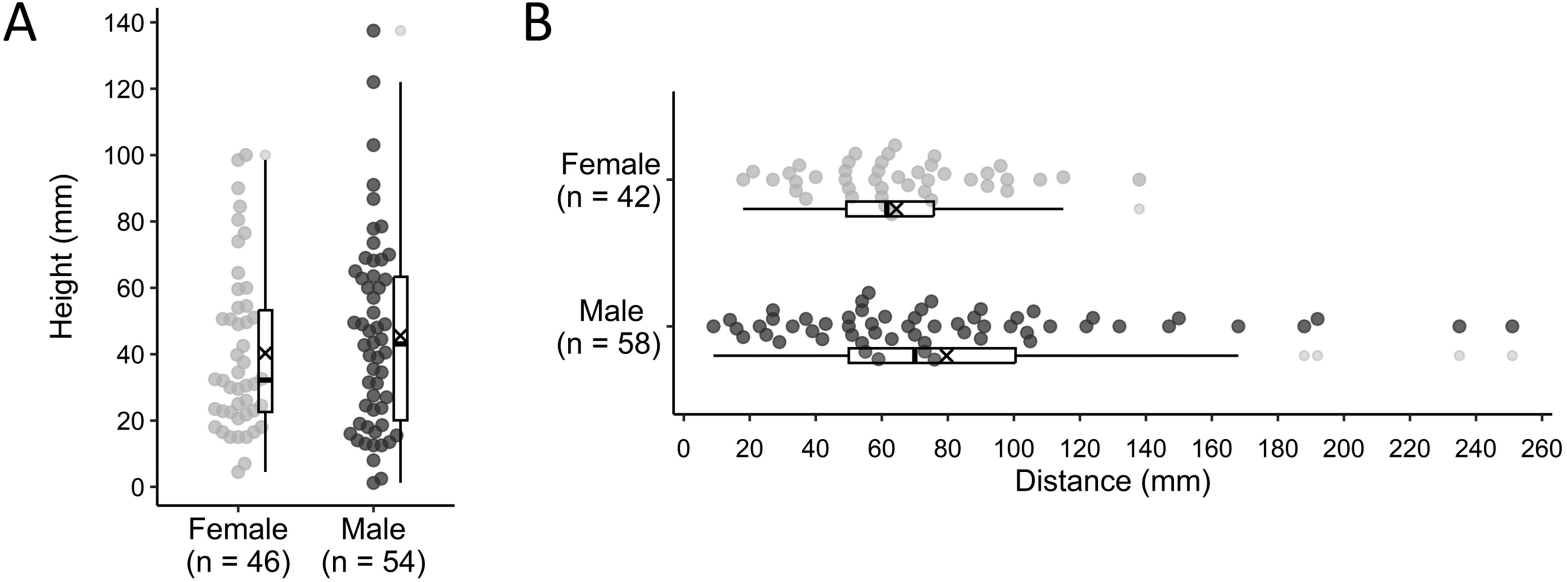
a) Jumping height of *Tunga penetrans*; b) jumping distance of *Tunga penetrans*. Crosses indicate mean values.

In the vertical height measurements, all the emerged adults were *T. penetrans* (*n* = 105). Among them, 100 adults stuck to the sticky tape: 46 females and 54 males. The remaining 5 adults were found at the bottom and therefore not recorded: 3 females and 2 males. There was no adult stuck on the top side. Female jumping heights ranged from 4.5 to 100 mm with a mean of 40 mm and male jumping heights ranged from 1.2 to 138 mm with a mean of 46 mm. Figure 3 shows the distribution of sand fleas’ jumping height. There was no significant difference between the vertical jumping heights of females and males (*P* = 0.34).

In the experiment assessing horizontal jumping distance, a total of 100 adults successfully emerged from the 119 cocoons: 42 females and 58 males. The minimum jumping distance record was 18 mm for females and 9 mm for males, and the maximum record was 138 mm for females and 251 mm for males (Fig. 3). The mean jumping distance was 64 mm for females and 80 mm for males (*P* = 0.06).

## Discussion

Depending on the species, fleas jump to a height of 50–100 times their body size, and to a maximum of 100 times their size in a horizontal direction (Rothschild et al. 1973, Dryden and Broce 1993, Cadiergues et al. 2000, Krasnov et al. 2003). We showed that the same applies for both sexes of *T. penetrans*, and hence *T. penetrans* has the same jumping capacity relative to their body size as its larger relatives. Whilst not reaching statistical significance at 0.05 level, males demonstrated more frequently superior jumping capabilities in terms of both height and distance. This observation can be attributed to the slightly greater body size of males relative to females of *T. penetrans.*

We would argue that the preferred location for female fleas to burrow, the feet, is not due to the limited jumping ability of *T. penetrans* but might reflect an evolutionary adaptation to the human host’s behavior that would maximize reproduction success. It has been observed that female sand fleas run over the skin in search of appropriate places to embed themselves after jumping onto hosts (Eisele et al. 2003, Elson et al. 2022). The location of a host and the location of the most suitable sites on the body to embed into the skin are likely guided by biologically programmed preferences based on semiochemicals; however, to date, no studies have been implemented to investigate this. Since the female sand flea is parasitic and does not leave the host to lay eggs, there is a need to increase the chance of eggs dropping on the ground when they get expelled from the embedded female fleas. The parasite’s position at the feet or along the hoof coronary band in clawed animals (Mutebi et al. 2016) will maximize the chance of the off-host stages developing in their required environment in the upper layer of soil. Other sessile female fleas of the familyTungidae (Siphonatera) like *T. penetrans* are reported to have host-species-specific attachment sites, for example: the top head of bats, *Hectopsylla pulex* (Haller, 1880) *?*; rodent ears, *Tunga caecata* (Enderlein 1901), *Tunga caecigena*, (Jordan & Rothschild 1921), *Tunga libis*, and (Smit 1962), *Tunga monositus* (Barnes & Radovsky 1969); the tail base of rodents, *Tunga bossi n. sp.*; the ventral abdomen, *Tunga travassosi*, (Pinto & Dryfus 1927), *Neotunga euloidea* (Smit 1962); the skin around anus, *Tunga callida*; toes, n. sp., *T. trimamillata* and *Tunga hexalobulata* n. sp. (Linardi and de Avelar 2014, Linardi et al. 2014). Besides reproductive reasons, it is speculated that these predilection sites are caused by a combination of (i) sites with regular contact with soil, (ii) sites where the host cannot easily remove the parasite by grooming, (iii) the structure of hair and thickness of fur (Rothschild 1992, Linardi and de Avelar 2014), and possibly (iv) access to blood vessels. Still, what drives these specific preferences seems to be unclear. Investigation of cues guiding the location for parasitization might further elucidate their host-finding strategies and could guide the development of traps.

In terms of biosafety measures for laboratory experiments, it will be vital to take the jumping capacity of this organism into account and prevent escapes by installing sufficiently wide sticky traps (>300 mm) around experimental setups and door frames. In practical disease prevention, it might also be recommended to seal cracks on floors and walls to prevent hiding places; however, the relevance of such an intervention would require further studies of the sand flea’s behavior in its natural environment.

It is important to acknowledge that the measured jumping heights and distances do not denote the maximum a flea might have achieved since they were immediately stuck on the sticky tape somewhere along their trajectories. Nonetheless, the results debunk the myth that *T. penetrans* has limited jumping abilities and challenge the notion that their clustering on hosts’ feet is a result of this.

## Data Availability

Data are contained within the article.
